# Transcranial stimulation as a possible therapeutic proposal in long COVID

**DOI:** 10.3389/fresc.2026.1766757

**Published:** 2026-02-27

**Authors:** Lidiane Palheta Miranda dos Santos, Julliana Vieira Leão, Ketelen Yasmim Braga de Moraes Silva, Danilo Lameira dos Santos, Chrisllane Nascimento Batista, Jonna Almeida Barros, Alna Carolina Mendes Paranhos, Ápio Ricardo Nazareth Dias, Luiz Fábio Magno Falcão

**Affiliations:** 1 Centro de Ciências Biológicas e da Saúde, Universidade do Estado do Pará, Belém, Brazil; 2 Programa de Pós Graduação em Biologia Parasitária na Amazônia, Universidade do Estado do Pará, Belém, Brazil; 3 Faculdade de Medicina, Universidade de São Paulo, Belém, Brazil

**Keywords:** long COVID, neurological rehabilitation, neuromodulation, therapeutic interventions, transcranial stimulation

## Abstract

The COVID-19 pandemic triggered an unprecedented global health crisis, with significant repercussions on the mental and neurological health of millions of individuals. Long COVID, characterized by persistent and debilitating symptoms, including chronic fatigue, pain, cognitive impairment, and mood swings, represents a substantial therapeutic challenge. In this context, neuromodulation emerges as a promising therapeutic strategy, offering new perspectives for the management of refractory neurological symptoms. This article aims to critically review the current evidence on the use of neuromodulation in patients with long COVID.

## Introduction

According to the World Health Organization (WHO), the COVID-19 virus (SARS-CoV-2) has infected more than 765 million people worldwide (1, 2). SARS-CoV-2 is considered neuroinvasive, capable of reaching the nervous system through multiple pathways, including the olfactory nerve, bloodstream, and brainstem, resulting in neurological disorders (3). Although COVID-19 is primarily a respiratory infection, it may generate prolonged sequelae known as Long COVID, affecting both severely ill and non-hospitalized individuals (4, 5). This may be attributed to impairment of the blood–brain barrier, leading to increased glutamate levels, which in turn promote oxidative stress, endothelial dysfunction, and hypercoagulability. Additionally, reduced activity of the angiotensin-converting enzyme (ACE) caused by infection contributes to a hyperinflammatory state (6).

Persistent symptoms include fatigue, pulmonary dysfunction, anosmia, cognitive alterations, among more than 50 long-term effects (4), Long COVID is a multifaceted clinical entity affecting a substantial proportion of individuals following SARS-CoV-2 infection (4, 5). Neurological manifestations include headache, chronic fatigue, neuropathic pain, cognitive impairment, anosmia, ageusia, and mood changes, which interfere with occupational performance, memory, concentration, and overall quality of life (6, 7).

Neuromodulation has been proposed as a therapeutic option to manage neurological symptoms (6, 8, 9). Recent evidence indicates that non-invasive neuromodulation may contribute to improvements by modulating inflammatory processes and neuroplasticity (10–19). tDCS may reduce persistent neuroinflammation by modulating microglial activation and cytokine levels. By attenuating the inflammatory milieu, this technique creates more favourable conditions for neural plasticity, while the active promotion of new synaptic connections contributes to the functional compensation of deficits. Thus, tDCS emerges as an adjuvant intervention targeting the core pathophysiological mechanisms of long COVID (18, 19, 21).

Thus, neuromodulation, a rapidly expanding field aimed at modulating neural activity through non-invasive or minimally invasive techniques, emerges as an innovative therapeutic approach to mitigate persistent neurological symptoms.

## Current evidence on neuromodulation in long-term COVID

Neuromodulation is an expanding field that modulates neural activity through non-invasive or minimally invasive techniques and has emerged as a promising therapeutic option to mitigate persistent neurological symptoms (23, 25, 26). Therapeutic modalities include: Transcranial Magnetic Stimulation (TMS): uses magnetic fields to induce electrical currents in the brain, modulating cortical excitability; Transcranial Direct Current Stimulation (tDCS): Applies direct electrical current to the scalp, altering neuronal polarity and cortical excitability (18, 19).

Although literature is still limited, preliminary studies suggest that neuromodulation may reduce neurological symptoms in long COVID patients (Table 1). For instance, TMS and tDCS have demonstrated improvements in fatigue, pain, and cognitive function. Studies highlight the importance of integrating immunology, neurology, psychiatry, and engineering to develop clinical strategies using non-invasive neuromodulation for treating COVID-19 sequelae, offering a safe and accessible treatment option (15, 20). Recent evidence indicates significant advances in long-term COVID rehabilitation. A meta-analysis of 51 randomized clinical trials with 4,026 participants showed that interventions such as physical training, respiratory muscle strengthening, and telerehabilitation improve functional capacity, cardiorespiratory performance, reduce dyspnea and fatigue, and enhance quality of life. However, no consistent effects were observed on spirometric parameters (FEV1, FVC, FEV1/FVC), global fatigue (MFIS), or anxiety and depression symptoms (8).

**Table 1:** Primary studies cited in manuscript.

Author	Year	Type of study	Study population	Method	Primary outcome
Santana et al. (21)	2023	Randomized, double-blind, placebo-controlled clinical trial	Patients aged 18–80 years with a diagnosis of PASC-related fatigue.	HD-tDCS 4 × 1 with a central electrode over M1; 30-s ramp-up and ramp-down; 3 mA intensity; 10 sessions of 30 min; rehabilitation program conducted in parallel with the stimulation.	Significant reduction in fatigue assessed by the MFIS was found when the active group was compared with the sham group.
Andrade et al. (22)	2022	Randomized, double-blind, placebo-controlled clinical trial	Patients aged 18 years or older, diagnosed with SARS-CoV-2 by PCR, and undergoing weaning after receiving at least 48 h of mechanical ventilation.	HD-tDCS 4 × 1 with a central electrode over M1; 30-s ramp-up and ramp-down; 3 mA intensity; two sessions per day for 10 consecutive working days; 30-min duration per session; pulmonary rehabilitation conducted in parallel with the stimulation.	The active group showed more ventilator-free days during the first 28 days compared with the sham group.
Oliver-Mas et al. (29)	2023	Randomized, double-blind, placebo-controlled pilot study	Patients diagnosed with SARS-CoV-2 by PCR and reporting fatigue symptoms.	tDCS with the anode over the left DLPFC and the cathode over the contralateral supraorbital region; 15-s ramp-up and ramp-down; 2 mA intensity; 8 consecutive sessions, one per day on weekdays; 20-min duration per session.	The active tDCS group was associated with improvement of fatigue symptoms at the end of treatment and at 1-month follow-up compared with the sham group.
Oliver-Mas et al. (30)	2025	Randomized controlled trial	Post-COVID patients with PCR-confirmed COVID-19 at least 6 months before study inclusion and self-reported fatigue symptoms.	tDCS with the anode over the left M1 or DLPFC and the cathode over the contralateral supraorbital region; 10-s ramp-up and ramp-down; 2 mA intensity; 15 sessions, one per day on weekdays; 20-min duration per session; use of CT in parallel with the sessions.	The M1 + CT group showed a slight improvement in fatigue symptoms and sleep quality compared with the DLPFC + CT group. Overall, both groups showed improvements in cognition and sleep quality with reduction of anxiety, depression, pain,
Vidal et al. (19)	2025	Randomized, double-blind, placebo-controlled clinical trial	Patients aged 18–75 years with a diagnosis of COVID-19 within the last 6 months and incident cognitive symptoms persisting for 4 weeks or more after infection.	tDCS with the anode over the left side (F3) and the cathode over the right side (F4) over the prefrontal cortex; 30-s ramp-up and 45-s ramp-down, respectively; 2 mA intensity for the active protocol and 1 mA for the sham protocol; 20 consecutive sessions on weekdays; 20-min duration per session; use of CT in parallel with the sessions.	The tDCS + CT group showed significant improvements in inhibitory control, processing speed, and divided attention assessment tests compared with the sham group.
Souza et al. (11)	2024	Case Series	Six patients with COVID-19 infection confirmed by a nasal swab test, with an acute infection duration of 2–6 months. Patients reported dyspnea, cough, odynophagia, and rhinorrhea.	HD-tDCS 4 × 1 with a central electrode over M1; 30-s ramp-up and ramp-down; 3 mA intensity; 10 sessions; 20-min duration per session; IMT conducted in parallel with the sessions.	Patients improved their SMIP at the end of treatment, as well as respiratory muscle function, inspiratory muscle power, and pulmonary function.
Gómez et al. (14)	2021	Case Report	A 58-year-old patient with PCR-confirmed COVID-19 and signs of fatigue and anxiety.	tDCS with the anode over the left side (F3) and the cathode over the right side (F4) over the prefrontal cortex; 3 mA intensity; 20 sessions, one per day, each lasting 20 min.	A clinically significant reduction in HARS, HDRS, and MFIS scores.
Klírová et al. (20)	2024	Randomized, double-blind, placebo-controlled study	Patients aged 18–75 years; symptom duration longer than 1 month after infection; a score ≥ 40 on the FIS questionnaire; presence of PASC-related neuropsychiatric symptoms.	tDCS with the anode over the left side (F3) and the cathode over the right side (F4) over the prefrontal cortex; 30-s ramp-up and ramp-down; 2 mA intensity; 20 sessions, one per day on weekdays; 30-min duration per session.	Change in FIS score at the end of treatment in both groups, being higher in the sham group, but with no statistically significant difference.

MFIS, modified fatigue impact scale; PASC, post-acute sequelae of SARS-CoV-2 infection; PCR, polymerase chain reaction; MV, mechanical ventilation; M1, primary motor cortex; DLPFC, dorsolateral prefrontal cortex; CT, cognitive training; IMT, inspiratory muscle training; SMIP, sustained maximal inspiratory pressure; HARS, Hamilton anxiety rating scale; HDRS, Hamilton depression rating scale; FIS, fatigue impact scale.

Combining rehabilitation with neuromodulation has shown promise (19). Adding tDCS has been associated with a significant reduction in the physical component of fatigue in individuals with long COVID (21). Similarly, a case report demonstrated that tDCS contributed to reducing anxiety and depression symptoms in post-COVID patients, supporting the potential role of neuromodulation as an adjunctive therapy in rehabilitation (15, 17).

### Transcranial direct current stimulation and fatigue

A randomized, double-blind clinical trial conducted in Paraíba involving 70 patients with fatigue related to chronic post-COVID syndrome demonstrated that high-definition tDCS (HD-tDCS), applied over the left motor cortex (M1) for 10 sessions over 5 weeks, significantly reduced fatigue as measured by the Modified Fatigue Impact Scale (MFIS) (19, 21), including cognitive and psychosocial dimensions, compared to the sham group. Active stimulation involved 3 mA for 30 min, while the placebo group received only ramp-up and ramp-down currents without sustained stimulation. Both groups also participated in individualized rehabilitation programs based on post-COVID guidelines, including breathing exercises, gradual stretching, and submaximal resistance training, with progression guided by the Borg scale and fatigue symptoms (19, 20).

Similarly, a randomized clinical trial with 56 adults who had undergone mechanical ventilation for at least 48 h and presented moderate to severe acute respiratory distress syndrome (ARDS) demonstrated that HD-tDCS applied over the left diaphragmatic primary motor cortex in a 4 × 1 montage significantly increased ventilator-free days within the first 28 days compared to controls. The protocol consisted of 3 mA for 30 min, twice daily for 10 weekdays, with systematic electrode repositioning (22, 23). Both groups received concurrent pulmonary rehabilitation, including inspiratory muscle training starting at 30% of maximal inspiratory pressure with 10% daily progression, physiotherapy support, and supplemental oxygen as needed (22).

Additional evidence from a study involving 47 post-COVID patients with persistent fatigue (>6 months), mostly middle-aged women, investigated the effects of eight sessions of tDCS over the left dorsolateral prefrontal cortex (2 mA for 20 min) in a double-blind, placebo-controlled design over 2 weeks. Active stimulation significantly reduced physical fatigue both immediately post-treatment and at 1-month follow-up, alongside a reduction in depressive symptoms. Cognitive fatigue and quality of life did not show significant improvements. Adverse effects were mild, confirming good tolerability (23). Furthermore, a case series of six patients with persistent respiratory sequelae post-COVID investigated the combination of HD-tDCS with inspiratory muscle training (IMT). The integrated intervention enhanced respiratory muscle strength and efficiency, increased inspiratory capacity, ventilation, lung volumes, and maximal inspiratory pressure (SMIP). Although preliminary, this approach demonstrated potential as a safe and effective strategy for long COVID respiratory rehabilitation, with the need for larger studies to validate findings (25).

**Figure 1:**
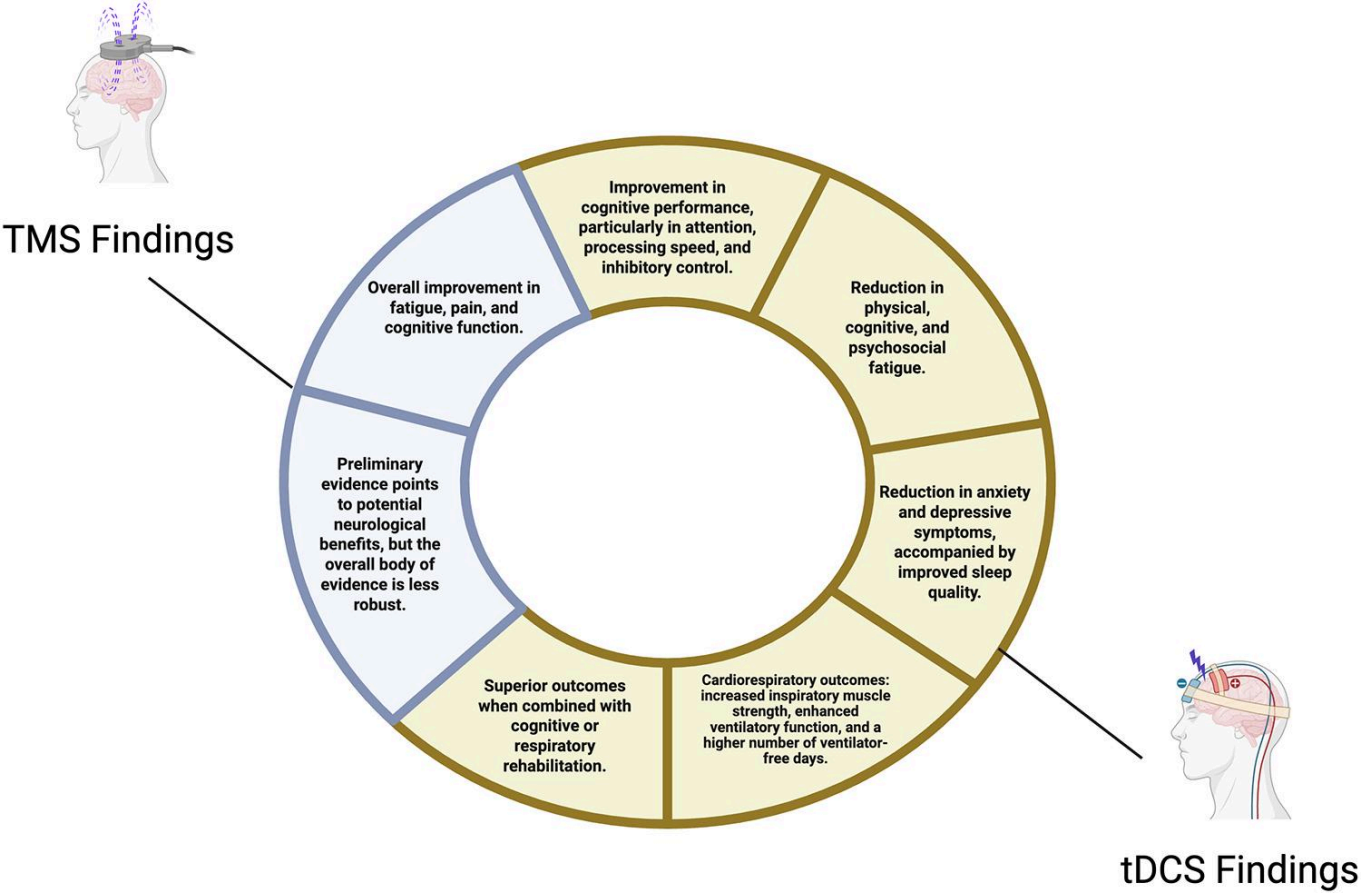
Effects of brain stimulation in neurological disorders.

Overall, these studies converge on the central point that neuromodulation, particularly HD-tDCS, is a promising adjunct in rehabilitating persistent post-COVID symptoms, including fatigue and respiratory performance. While populations, protocols, and objectives differed, the combination of brain stimulation with structured rehabilitation consistently produced superior outcomes compared to conventional therapies alone. HD-tDCS shows good tolerability, with adverse events limited to mild and transient skin discomfort, positioning it as a non-pharmacological, scalable therapeutic option in post-COVID rehabilitation (20, 23–25) (Figure 1).

### Transcranial direct current stimulation and cognition

A randomized study evaluated the efficacy of tDCS combined with cognitive training in individuals with persistent post-COVID symptoms, including fatigue and cognitive impairments persisting on average for almost 3 years after infection (26, 27). Participants, predominantly middle-aged women, were randomly assigned to two groups: one received tDCS applied to the primary motor cortex (M1) and the other to the dorsolateral prefrontal cortex (DLPFC). Both groups underwent 15 sessions accompanied by structured cognitive training. Pre-intervention, immediate post-intervention, and 1-month follow-up assessments showed consistent improvements across multiple domains, including fatigue, cognitive performance, anxiety, depression, pain, and sleep quality. Stimulation of M1 showed slight superiority in reducing fatigue and improving sleep, suggesting the potential of combining neuromodulation with cognitive training as a safe and promising approach for persistent post-COVID symptoms (27).

In a pilot study, tDCS applied to the prefrontal cortex combined with structured cognitive training was investigated in adults with persistent cognitive complaints post-COVID. Participants, mostly women with an average age of 43 years, were randomized to 20 sessions of active or sham tDCS, both paired with the same cognitive training. Neuropsychological assessments demonstrated modest but significant improvements in inhibitory control, processing speed, and divided attention. Both groups also showed reduced anxiety and depressive symptoms, highlighting an emotional benefit associated with cognitive training. The authors emphasized the need for larger, methodologically robust studies to confirm these effects and define the role of tDCS in managing cognitive sequelae of long COVID (28).

A randomized, double-blind, placebo-controlled study evaluated tDCS targeting the medial prefrontal cortex for neuropsychiatric symptoms in long COVID. Participants were assigned to active tDCS or sham groups, receiving 20 sessions of 30 min each. The study did not find significant improvements in cognitive function, suggesting that tDCS monotherapy may be insufficient. Limitations included small sample size and possible suboptimal stimulation protocol (29).

Another study investigated non-invasive neurostimulation for persistent anosmia post-COVID in two phases. In the first phase, 25 participants underwent olfactory assessment and EEG recordings to identify target regions. In the second phase, 15 participants continued with neurostimulation divided into active and sham groups. Stimulation targeted olfactory structures (olfactory bulb, tract, and piriform cortex) across five 20-minute sessions. Participants with more severe initial olfactory deficits showed better outcomes, though statistical significance was limited due to small sample size (30). Across these four studies, populations were predominantly female, with mean ages between 43 and 47 years and persistent symptoms approximately 32 months post-infection. Results were heterogeneous. Studies combining tDCS with cognitive training (27, 28) showed significant improvements in fatigue, cognition, anxiety, depression, and sleep quality, whereas tDCS monotherapy or olfactory stimulation alone (29, 30) had limited or selective effects. Methodological limitations included small sample sizes, heterogeneity in protocols, and variable stimulation targets, which hindered direct comparison and generalizability.

Overall, tDCS paired with cognitive training emerges as a promising approach for managing cognitive deficits in post-COVID-19 patients. However, larger, rigorously designed clinical trials with standardized protocols are required to determine optimal parameters and validate clinical efficacy (27–30) (Figure 1).

## Challenges and limitations

The application of neuromodulation in long COVID faces substantial challenges, including the heterogeneity of symptoms, the lack of complete understanding of the underlying pathophysiological mechanisms, and the need for personalized treatment protocols. Furthermore, the accessibility and cost of neuromodulation techniques may limit their widespread use.

### Future directions

To advance the use of neuromodulation for long COVID, well-designed clinical studies are needed to assess efficacy, safety, and best treatment protocols. Furthermore, it is crucial to investigate the mechanisms underlying long COVID and how neuromodulation can influence these processes. Neuromodulation presents itself as an innovative and promising therapeutic approach to alleviate the debilitating neurological symptoms of long COVID. While current data are encouraging, there is an urgent need for more research to establish efficacy, safety, and best treatment protocols. With increased understanding of long COVID and advancements in neuromodulation techniques, there is hope that these approaches can offer significant relief to the millions of patients affected by this condition.
